# Association between lifestyle choices and mental health among medical students during the COVID-19 pandemic: A cross-sectional study

**DOI:** 10.1371/journal.pone.0274525

**Published:** 2022-10-07

**Authors:** Hela Ghali, Rim Ghammem, Imen Baccouche, Mouna Hamrouni, Nada Jedidi, Hichem Smaali, Souha Earbi, Balkis Hajji, Amina Kastalli, Hind Khalifa, Khalil Bilel Maagli, Rawia Romdhani, Heni Halleb, Farouk Jdidi

**Affiliations:** 1 Faculty of Medicine of Sousse, University of Sousse, Sousse, Tunisia; 2 Department of Prevention and Security of Care, Sahloul University Hospital, Sousse, Tunisia; 3 Department of Epidemiology, Farhat Hached Hospital, «LR19SP03», Sousse, Tunisia; King Saud University, SAUDI ARABIA

## Abstract

**Introduction:**

Mental health is recognized as a critical component of public health Given the close relationship between mental health and life style and the importance of students as valuable human resources, the present study aimed at determining the relationship between life style and mental health among medical students in Sousse during COVID-19 pandemic.

**Methods:**

We conducted a cross-sectional observational study in university students from the first to the fifth grade of the Faculty of Medicine of Sousse during the academic year 2020/2021. Data were collected anonymously via an online questionnaire published on the Facebook groups of each grade, on december 2020. The online survey consisted of three sections. The first one aimed to collect sociodemographic information. The second section of the survey addressed recent lifestyle choices and the third one assessed psychological distress using the French version of 12 items of The General Health Questionnaire (GHQ-12). The significance level was considered 0.05.

**Results:**

Overall 147 were studied. Using the bimodal scoring, the total score was between 0 and 12, to evoke psychiatric disorders, we set the threshold of 4. For our sample, the median scale was 7 [4-9], and more than half of the students (68%; n = 100) had a score higher than 4. Psychiatric disorder was significantly more frequent in female students (73.3% vs 42.3%; p = 0.002). Higher GHQ-scale was found in younger students, foreigners, students who need more than 30 minutes to get to the faculty, unemployed students, and students of fifth grade. However, differences were not statistically significant. Regarding lifestyle choices, we found that physical inactivity, no smoking habits, no alcohol use, no illicit substance use, other substance use, changing in eating habits, and absence of coping methods of stress were higher in students with psychiatric disorders. However, this association was statistically significant only for physical activity (p = 0.016). The results of the regression analysis suggest female gender as an independent predictor of high GHQ-12 scores. Practicing physical activity was found as protective factor for psychiatric disorders.

**Conclusion:**

Considering the vital role of medical students in providing and promoting community health, the need for more detailed planning and interventions to improve their life style and mental health is essential.

## Introduction

Mental health is recognized as a critical component of public health [1]. The World Health Organization (WHO) describes mental health as a “state of well-being in which every individual realizes his or her own potential, can cope with the normal stresses of life, can work productively and fruitfully, and is able to make a contribution to her or his community” [2]. Similarly, mental health is an essential component of healthy living, and vice versa.

Furthermore, mental health is a requisite for the sustainability of social, occupational, and academic performance of people in society [3, 4].

Leading a healthy life is beneficial for one’s well-being. According to the WHO, a healthy lifestyle means regular physical activity, abstaining from smoking, limiting alcohol consumption, eating well to prevent overweight and non-communicable diseases. Such a lifestyle will have benefits on mental health. [5–7].

The full impact of the COVID-19 pandemic on health, economy, interpersonal relations, and daily habits has resulted in changing in individuals’ wellbeing, mental health and lifestyle-related behaviors [8, 9].

Thus, it is a truism to state that the Covid-19 pandemic had a major impact on our society, in Tunisia as elsewhere, in terms of health, economy and professional, family and social life [10, 11]. This impact has manifested itself as a sleep disorder and psychological disorders according to some studies [12]. Young people are particularly vulnerable to the disruptions caused by the pandemic, and many are being left behind in education, economic opportunities, health and well-being during a crucial stage of their development and education [13]. All these changes can lead to dangerous behaviors such as suicidal ideation [14, 15].

As the epidemic continues to spread, authorities have been forced to tighten isolation measures and close all schools and universities [8]. In Tunisia, since the 1^st^ of March 2020, all schools, colleges, and universities indefinitely suspended their attendance, and, where possible, activated online learning as an alternative.

Even before COVID-19, it was clear that university students are uniquely vulnerable to mental health problems [16]. In fact, the concern about academic performance coupled with relationship difficulties and feelings of loneliness make students susceptible to developing mental problems [8, 17]. According to the Turkish study on perceived stress during the COVID-19 outbreak, generalized anxiety disorder was found in 52% of students and depression in 63% [18].

Other dangerous behaviors, such as drug and alcohol consumption and even self-harm are frequently initiated during the lockdown [19]. Indeed, Canadian and Spanish studies showed that students’ high alcohol and tobacco consumption were significantly compounded during the pandemic [20, 21]. Additionally, the lockdown and staying at home will cause a change in lifestyle habits including the limitation of physical activity [8]. In fact, it was found that the current quarantine negatively affected the practice of physical exercise by the student population [22].

Moreover, constant mass-media reports about COVID-19 death rates lead to distress [23], and thus, people are more likely to choose less healthy ‘comfort foods’ [24, 25], smoke more cigarettes [26], and more frequently reach for alcohol [27]. Furthermore, during the lockdown, there is a substantial risk of increasing substance use [28].

Thus, students are at risk of developing mental health problems especially since they are already vulnerable and highly susceptible to addiction and psychological crises.

Most studies have focused on studying the impact of the pandemic on either the general population or medical and paramedical personnel. However, the impact on the student population requires more attention as students find their lifestyles totally changed [8].

According to Nilufar S et al, the mental health of medical students is associated with their lifestyle choices. Thus, changing it in a healthy way will improve their mental health [29]. According to Davarinejad O et al. [30], a statistically significant negative association was found between mental health scores and those assessing different lifestyle domains.

In this context, our study aimed to investigate lifestyle and behavioral choices experienced by Tunisian medical students due to the epidemiological situation and their association with mental health.

## Material and methods

### 1. Study design

We conducted a cross-sectional observational study in the Faculty of Medicine of Sousse during the academic year 2020/2021.

### 2. Study population

The survey was aimed at university students from the first to the fifth grade. Data were collected anonymously via an online questionnaire published on the Facebook groups of each grade, during one month on december 2020.

### 3. Data collection

The online survey consisted of three sections.

The first one aimed to collect sociodemographic information, including gender, age, residence, activities other than studies, income source, etc.

The second section of the survey addressed recent lifestyle choices that were a result of the pandemic : physical activity, eating habits (quantity, frequency of meals and type of food), tobacco use, alcohol use, illicit-substance use (cannabis and cocaine), other substance use (drugs, stimulant and energy drinks), and coping methods for stress defined as reading books on personnel development, practicing art activities, relaxation and attention techniques. These elements were assessed using questions where the answer was either yes or no.

While there is an on-going debate about the validity of self-reported alcohol consumption compared to objective data, recent studies conclude that self-report can reliably estimate alcohol consumption in low to moderate drinkers [31]. Such items are regularly used in epidemiological studies on alcohol consumption [32]. Current smoking was assessed using one item: “Do you smoke?” Answer categories were ‘no’, ‘yes’.

The last section of the survey included the questionnaire aimed to assess psychological distress using the French version of 12 items of The General Health Questionnaire (GHQ-12) [33, 34].

The 12-item General Health Questionnaire (GHQ-12) is a self-report measure of psychological morbidity, intended to detect psychiatric disorders in community settings and non-psychiatric settings. It is widely used in clinical practice, epidemiological research and for research in psychology. Due to its brevity, is probably the most popular and so, has been extensively evaluated in terms of its validity and reliability as a one-dimensional indicator of the severity of psychological morbidity.

The 12-Item General Health Questionnaire (GHQ-12) [35] consists of 12 items, each one assessing the severity of a mental problem over the past few weeks using a 4-point Likert-type scale (from 0 to 3). The score was used to generate a total score ranging from 0 to 36. The positive items were corrected from 0 (always) to 3 (never) and the negative ones from 3 (always) to 0 (never). High scores indicate worse health.

Three rating systems were proposed:

- Dimensional rating: Likert scale rated from 0 to 3, with 0 corresponding to the first modality, and 3 to the last modality.
- Dichotomous scoring 0-0-1-1: The 2 responses reflecting an improvement compared to the usual situation can be scored "0", while the 2 responses reflecting an aggravation will be scored "1".
- Dichotomous scoring, known as cGHQ: Positively worded items are scored 0-0-1-1 and negatively worded items are scored 0-1-1-1. This scoring allows for long-lasting conditions for which subjects may feel that their health status has not changed from usual.

Whatever the scoring system, the addition of the responses obtained for each item makes it possible to calculate an overall score considered to indicate psychological damage (or a high probability of psychological damage) when it is high. It is difficult to know which scoring system is best. The dichotomous 0-0-1-1 scoring system seems to be the most satisfactory according to Goderberg [36]. We used the bimodal scoring (scoring 0 for " better than usual" or "as usual" and 1 for " less well than usual" or "much less than usual"). Interpretation: 0–4: Normal subject 5–12: Existence of a psychiatric disorder.

### 4. Data analysis

Statistical analysis was performed using SPSS ver. 25.0 (IBM, Chicago, IL, USA).

Data are represented as number and percentage for categorical variables, and mean ±standard deviation or median and interquartile range in square brackets [IQR] for continuous variable. The Kolmogorov-Smornov test was performed in order to evaluate variables distribution.

Chi square test was used to compare categorical variables, and Student t-test to compare means. To study associations between sociodemographic characteristics, lifestyle choices and mental helath, binary logistic regression model was used. Variables at a p value ≤ 20% in the univariate analysis were the variables used in the model. The fit of the final model was evaluated with the Hosmer and Lemeshow test. Significant p value was set at 0.05.

### 5. Ethical approval

The research protocol was neither harmful to health nor patient safety or privacy. Furthermore, the study protocol obtained approval from the Ethical Committee of the University Hospital Farhat Hached in Sousse from Tunisia.

## Results

### 1. Participants’ characteristics

The studied group included a total number of 147 participants, within which 120 were female (81.6%) and 26 were male (17.7%). The mean age of the respondents was 21.62±1.9 years. Only 1.4% of the students were not of Tunisian nationality. Most of the participants were from Sousse (32.7%).

The highest response rate was noted among the fifth grade students (36.1%).

A detailed description of the demographic characteristics of the study group is presented in Table 1.

**Table 1:** Distibution of demographic characteristics of medical students, Sousse, Tunisia, 2021.

Characteristics	n	%
**DEMOGRAPHIC**		
**Gender**		
Male	26	17.8
Female	120	82.2
**Nationality**		
Tunisian	144	98.6
Other	2	1.4
**Place of residence**		
<100 Km	113	76.9
≥100 Km	34	23.1
**Travel time between faculty and residence**		
≤30 minutes	132	89.8
> 30 minutes	15	10.2
**Employement**		
Unemployed	112	76.2
Employed	35	23.8
**Source of income**		
Family	137	93.2
Scholarship	4	2.7
Family and scholarship	4	2.7
Self-supportive	2	1.4
**Housing during the academic year**		
University residence	13	8.8
With the family	63	42.9
Individuel rent	29	19.7
Collective rent	42	28.6
**EDUCATION**		
**Year (grade)**		
First	22	15.0
Second	22	15.0
Third	27	18.4
Fourth	23	15.6
Fifth	53	36.1
**Repeating the grade**		
Yes	16	10.9
No	131	89.1

### 2. Lifestyle factors

**Fig 1:**
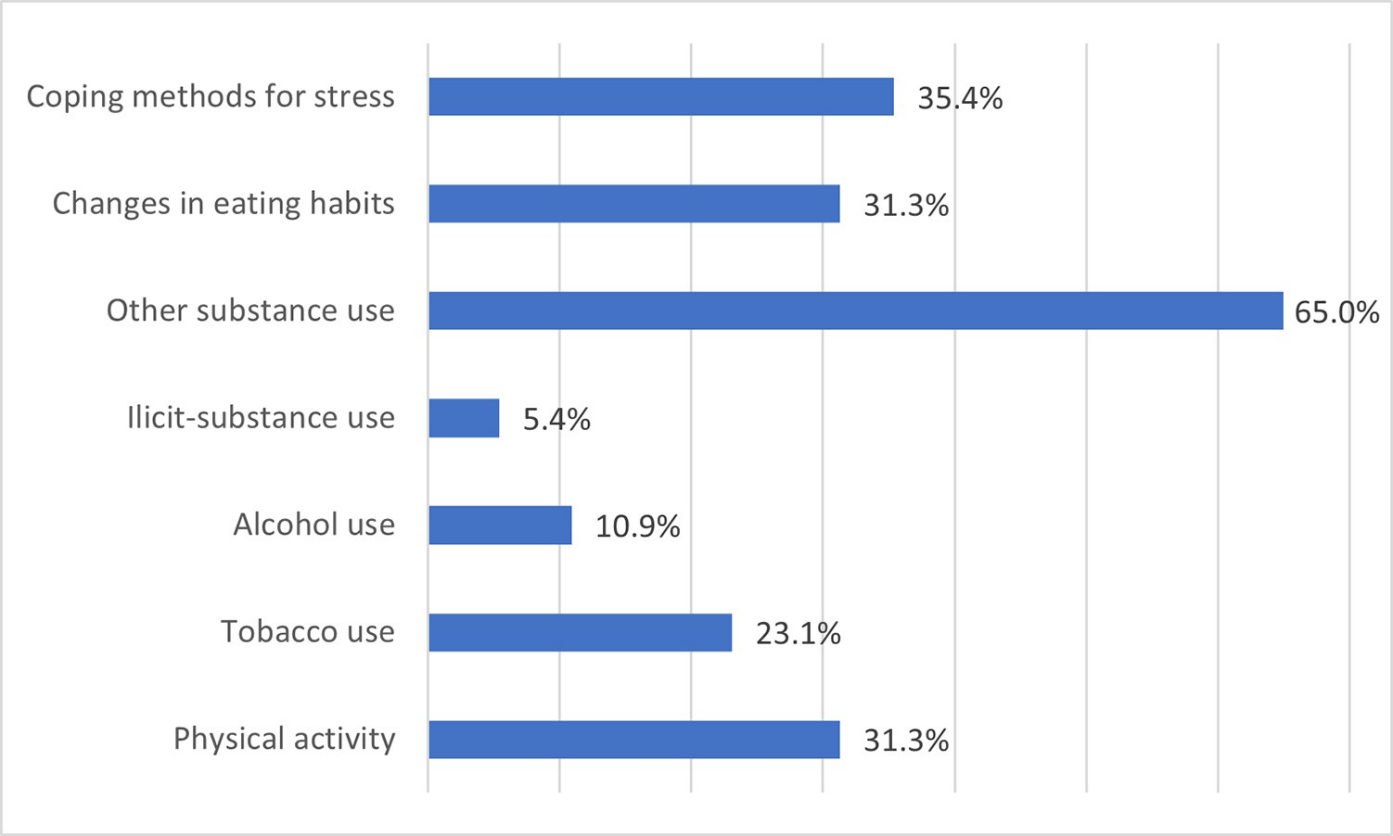
Descriptive values of lifestyle factors of the study sample, n = 147.

We assessed seven lifestyle factors which were : physical activity, changes in eating habits, tobacco use, alcohol use, illicit-substance use, other substance use, and coping methods for stress. Descriptive values of lifestyle factors are presented in Fig 1.

### 3. Mental health

Using the GHQ-12 scale, the mean scale was 19±6.45 with the extremes ranging from 5 to 35.

Distributions of students by GHQ-12 items are summarized in Table 2.

**Table 2:** Distribution by GHQ-12 items of medical students, Sousse, Tunisia, 2021.

Item	More than usual N (%)	As usual N (%)	Less than usual N (%)	Much less than usual N (%)
1. **Have you been able to focus on everything you do?**	54 (36.7)	62 (42.2)	21 (14.3)	10 (6.8)
2. **Did you miss sleep because of your worries?**	28 (19.0)	22 (15.0)	47 (32.0)	50 (34.0)
3. **Did you feel that you were playing a useful role in life?**	77 (52.4)	30 (20.4)	20 (13.6)	20 (13.6)
4. **Did you feel able to make decisions?**	74 (50.3)	35 (23.8)	12 (8.2)	26 (17.7)
5. **Did you feel constantly tense or under stress ?**	1 (0.7)	85 (57.8)	44 (29.9)	17 (11.6)
6. **Did you feel that you could not be able to overcome your difficulties?**	15 (10.2)	27 (18.4)	66 (44.9)	39 (26.5)
7. **Were you able to enjoy your normal daily activities?**	46 (31.3)	64 (43.5)	29 (19.7)	8 (5.4)
8. **Were you able to cope with your problems?**	80 (8.2)	43 (29.3)	12 (8.2)	12 (8.2)
9. **Have you been unhappy or depressed?**	12 (8.2)	43 (29.3)	64 (43.5)	28 (19.0)
10. **Have you lost confidence in yourself?**	41 (27.9)	23 (15.6)	49 (33.3)	34 (23.1)
11. **Did you consider yourself as someone who was worthless?**	61 (41.5)	18 (12.2)	47 (32.0)	21 (14.3)
12. **Did you feel relatively happy overall ?**	59 (40.1)	52 (35.4)	24 (16.3)	12 (8.2)

Using the bimodal scoring, the total score was between 0 and 12, to evoke psychiatric disorders, we set the threshold of 4.

For our sample, the median scale was 7 [4–9], and more than half of the students (68% ; n = 100) had a score higher than 4.

### 4. Factors associated with mental health : Univariate analysis

Psychiatric disorder was significantly more frequent in female students (73.3% vs 42.3% ; p = 0.002). Higher GHQ-scale was found in younger students, foreigners, students who need more than 30 minutes to get to the faculty, unemployed students, and students of fifth grade.

However, differences were not statistically significant (Table 3).

**Table 3:** Demographic characteristics associated with mental health in medical students, Sousse, Tunisia, 2021.

Characteristics	Psychiatric disosrder		OR CI_95%_	p-value
	No (n = 47) N (%)	Yes (n = 100) N (%)		
**DEMOGRAPHIC**				
**Gender**				
Male	15 (57.5)	11 (42.3)	3.7 [1.5–9]	**0.002**
Female	32 (26.7)	**88 (73.3)**		
**Age (mean±standard deviation)**	22±2.4	21.4±1.6	_	0.10
**Nationality**				
Tunisian	47 (32.6)	97 (67.4)	_	1
Other	0	**2 (100)**		
**Place of residence**				
<100 Km	35 (31)	**78 (69)**	_	0.63
≥100 Km	12 (35.3)	22 (64.7)		
**Travel time between faculty and residence**				
≤30 minutes	45 (34.1)	87 (65.9)	_	0.14
> 30 minutes	2 (13.3)	**13 (86.7)**		
**Employement**				
Unemployed	35 (31.3)	**77 (68.8)**	_	0.73
Employed	12 (34.3)	23 (65.7)		
**Source of income**				
Family	42 (30.7)	95 (69.3)	_	0.18
Scholarship	2 (50.0)	2 (50.0)		
Family and scholarship	1 (25.0)	**3 (75.0)**		
Self-supportive	2 (100)	0		
**Housing during the academic year**				
University residence	6 (46.2)	7 (53.8)	_	0.72
With the family	17 (27.0)	**46 (73.0)**		
Individuel rent	12 (41.4)	17 (58.6)		
Collective rent	12 (28.6)	30 (71.4)		
**EDUCATION**				
**Field of high school study**				
Mathematics	18 (34.6)	34 (65.4)	_	0.61
Experimental sciences	29 (30.5)	**66 (69.5)**		
**Year (grade)**				
First	9 (19.1)	13 (13.0)	_	0.23
Second	6 (12.8)	16 (16.0)		
Third	4 (8.5)	23 (23.0)		
Fourth	9 (19.1)	14 (14.0)		
Fifth	19 (40.4)	**34 (34.0)**		
**Repeating the grade**				
Yes	6 (37.5)	10 **(**62.5**)**	_	0.61
No	41 (31.3)	**90 (68.7)**		

Regarding lifestyle choices, we found that physical inactivity, no smoking habits, no alcohol use, no illicit substance use, other substance use, changing in eating habits, and absence of coping methods of stress were higher in students with psychiatric disorders.

However, this association was statistically significant only for physical activity (p = 0.016). (Table 4).

**Table 4:** Lifestyle choices associated with mental health in medical students, Sousse, Tunisia, 2021.

Lifestyle choices		Psychiatric disosrder		OR CI_95%_	p-value
		No (n = 47) N (%)	Yes (n = 100) N (%)		
**Physical activity**	Yes	21 (45.7)	25 (54.3)	0.14 [0.19–0.85]	**0.016**
	No	26 (25.7)	**75 (74.3)**		
**Tobacco use**	Yes	11 (32.4)	23 (67.6)	_	0.95
	No	36 (31.9)	**77 (68.1)**		
**Alcohol use**	Yes	8 (50.0)	8 (50.0)	_	0.10
	No	39 (29.8)	**92 (70.2)**		
**Ilicit-substance use**	Yes	3 (37.5)	5 (62.5)	_	0.70
	No	4 (31.7)	**95 (68.3)**		
**Other substance use**	Yes	28 (28.9)	**69 (71.1)**	_	0.26
	No	19 (38.0)	31 (62.0)		
**Changes in eating habits**	Yes	11 (23.9)	**35 (76.1)**	_	0.15
	No	36 (35.6)	65 (64.4)		
**Coping methods for stress**	Yes	19 (36.5)	33 (63.5)	_	0.38
	No	28 (29.5)	**67 (70.5)**		

### 5. Factors associated with mental health : Multivariate analysis

A logistic regression was performed to predict GHQ-12 as dependent variable, in relation to sociodemographic characteristics and the lifestyle choices items. The results of the regression analysis suggest female gender as an independent predictor of high GHQ-12 scores. However, practicing physical activity was found as protective factor for psychiatric disorders (Table 5).

**Table 5:** Lifestyle choices associated with mental health in medical students: Multivariate analysis.

Factor	Adjusted-OR	CI_95%_	p-value
**Female gender**	3.67	1.49–8.96	0.004
**Practicing physical activity**	0.42	0.20–0.91	0.028

## Discussion

The results of a systematic review carried out to assess the mental health of medical students in Asian countries indicated that 11% of the medical students were suffering from depression, while the prevalence of depression was higher in students in the preclinical level and students residing at home [37]. In another systematic review conducted to analyze the mental health of medical students in Brazil, it was reported that the prevalence of depression and anxiety in the students was 30.6% and 46.1%, respectively [38].

Lifestyle plays an important role in determining certain medical and psychiatric conditions. Indeed, a healthy lifestyle (eating healthy foods, getting more physical activity, quitting smoking, avoiding alcohol and illicit substances), will improve these conditions in a positive way [39].

The current COVID-19 pandemic have changed life satisfaction and mental health. The current study investigated lifestyle and behavioral changes experienced by Tunisian medical students due to the epidemiological situation and their effect on their mental health.

A lot of studies have shown that medical students are more prone to depression, more tired, and more prone to emotional exhaustion, depersonalization, and burnout syndrome than other students. Likewise, symptoms of depression, suicidal ideas, and dissatisfaction with personal accomplishments show the greatest prevalence during medical studies, while it subsequently declines in a physician’s career [40].

Many Polish students confirmed the neglect of their daily lives in relation to experiencing significant psychological distress related to the pandemic [8]. In accordance with our results, that suggest that the ability to maintain a physical activity and using coping methods of stress were related to lower psychological distress, regularizing and maintaining daily routines has been previously mentioned as a protective factor during the COVID-19 pandemic [41].

In fact, maintaining a daily routine as a protective factor is addressed in several studies, for exemple, on post-traumatic stress during natural disasters, [42] or populations living in high-stress environments, like forced immigrants [43].

Furthetmore, some early studies on eating behaviors amidst the COVID-19 crisis have already reported changes in food intake as a result of the pandemic [44–48].

The results of our study indicate that having received other substances such as other medications stimulant and energy drinks was related to higher psychopathological distress.

Another interesting, however concerning finding is, that higher psychological distress was found in students who do not smoke, not drink alcohol, neither use illicit substances.

Indeed, our findings suggest that, although only a small percentage of the studied population reported consumption of tobacco, alcohol and illicit substances, they were not significantly related to a higher psychopathological distress.

A study by Lechner et al. [49] conducted among college students at the onset of the pandemic and campus closure, reported an increase in alcohol consumption with an additional finding that those students who reported depression or anxiety demonstrated a higher increase in alcohol consumption. An explanation for why only 10.9% of the studied group reported consumption of alcohol could be related to the fact that most of respondents lived either with their families or in dormitories were they are not free. On the other hand, the absence of student gatherings following the closure of the universities may explain the considerable reduction in opportunities for alcohol consumption [8]. This hypothesis is also supported by the aforementioned study [49], where the authors have found that an increase in the consumption of alcoholic drinks was accompanied by an increase of drinking occasions overall, which at that time in Tunisa was unlikely.

In the study conducted by Davarinejad, et al [30], a positive and significant correlation was found between mental health and life style dimensions so that the variables of psychological health, social health, exercise, and health and environmental health were the best predictors of mental health. Also, in the study of Esfahani et al. [50], who studied mental health with an Islamic life style, reported a positive and significant correlation between the two categories.

According to our findings, coping methods of stress was related with mental well-being. In fact, theWorld Health Organization (WHO) defines life skills as “abilities for adaptive and positive behavior, that enable individuals to deal effectively with the demands and challenges of everyday life.” [51]. It has been noted that those who find themselves able to adapt to this changed circumstances have better mental health.

On the other hand, we noted that those who maintained physical activity, good stress management and an unmodified diet had better mental health.

The results of our multivariate analysis identified only two independent variables significantly associated with the mental health of our students which were female gender and physical activity.

Thus, it can be said that the mental health of medical students may be partly influenced by their lifestyle choices. As such, it is recommended that their behaviours be addressed to improve their mental health.

The results of this study should be generalized to other Tunisian universities with caution due to its small sample size, and thus, the present sample does not have good representativeness.

In fact, despite the fact that we solicited and encouraged students to participate in our study, the response rate remains low, and we couldn’t reach directly the students because of the sanitary situation.

In spite of all this, our study is the first, as far as we are aware, to analyze the mental health of medical students during the lockdown in relation to their lifestyle choices. Thus, a national multicenter study on a generalizable sample of selected universities is recommended.

## Conclusion

Among the repercussions of the COVID-19 pandemic, we can note the switch of university education to the virtual one, which has generated as a consequence the modification of the students’ lifestyle. However, the impact of this new lifestyle on mental health remains to be studied. Based on this observation, we interviewed students of the Faculty of Medicine of Sousse about the changes in their life choices and the potential alterations of their psychological state.

Students reported changing in their eating habits, lack of physical activity and use of both illicit substances and other substances such as drugs and energetic drinks. These changes were accompanied by a psychiatric disorder, although this association was significant for only lack of physical activity and female gender.

Thus, there are many factors that can influence the relationship between lifestyle and mental health. Therefore, special attention should be given to students in general and medical students in particular to improve overall health in the community.

## Supporting information

S1 File (SAV)
